# A111 CLINICAL AND PATHOLOGICAL OUTCOMES BETWEEN WATER EXCHANGE AND AIR INSUFFLATION USING NON-SEDATED EXTENDED FLEXIBLE SIGMOIDOSCOPY FOR AVERAGE RISK COLORECTAL CANCER SCREENING: PRELIMINARY FINDINGS FROM A RANDOMIZED CONTROL TRIAL

**DOI:** 10.1093/jcag/gwac036.111

**Published:** 2023-03-07

**Authors:** A Aulakh, B Parker, B Sullivan, M Recsky, C Oliveira, W Richardson, P Hirschkorn, R Perini, A Bak

**Affiliations:** 1 Faculty of Medicine , University of British Columbia; 2 Medicine , The Kelowna General Hospital , Kelowna; 3 Provincial Health Services Authority, Vancouver; 4 Division of General Surgery, The Kelowna General Hospital , Kelowna; 5 Faculty of Medicine , University of British Columbia , Victoria; 6 Medicine , University of British Columbia; 7 Division of Gastroenterology, The Kelowna General Hospital , Kelowna, Canada

## Abstract

**Background:**

Colorectal cancer (CRC) is Canada's third most common cancer type and represents approximately 11% of all cancer deaths. While sedated colonoscopy or flexible sigmoidoscopy (FS) continues to be considered for CRC screening, these modalities have limitations and risks. Another unevaluated screening modality, known as an extended FS (EFS), attempts to capitalize on the benefits of a FS while minimizing the risks involved with a sedated colonoscopy. EFS provides a scope-based examination up to the splenic flexure and then attempts to examine beyond, often to the point of the caecum. Providing the option for EFS may produce improvements in the patient experience and performance, which may improve the feasibility of using scope-based screening more broadly in screening programs.

**Purpose:**

To determine the extent non-sedated EFS using the water exchange method (WE) is associated with a complete colon examination compared to the traditional air insufflation (AI) method using CO2 in an average-risk screening population.

**Method:**

This randomized control trial included 90 non-sedated participants, screened by trained general surgery and gastroenterology clinicians at Kelowna General Hospital, British Columbia, Canada, using two different scope insufflation techniques, WE and AI. The primary outcome of interest was the cecal intubation rates (CIR), while secondary outcomes included the adenoma detection rate (ADR) and reported pain scores. Other metrics, such as patient satisfaction rates, sessile serrated adenoma detection rates (SSADR), and serrated lesion detection rates (SLDR) were also recorded.

**Result(s):**

The demographic characteristics between the WE and AI groups were statistically similar, with the mean age of participants being 58 and 57, respectively. During the study period, four endoscopists performed the EFS. There were higher initial satisfaction rates in the WE group vs the AI (95% vs 77%, satisfaction of ≥ 9/10 p = 0.028). CIR and ADR were similar between the WE and AI group (CIR = 93% vs 91%, p = 0.710), (ADR = 40% vs 34%, p = 0.660). The SSADR and SLDR were also similar between the WE and AI group (SSADR = 21% vs 14%, p = 0.408), (SLDR = 42% vs 36%, p = 0.528).

**Conclusion(s):**

EFS without sedation using either technique exceeds quality benchmarks recommended for sedated screening colonoscopy while maintaining adequate patient safety and comfort. The WE method optimizes a patient's overall experience making a strategy of average risk colorectal cancer screening with non-sedated WE EFS feasible.

**Please acknowledge all funding agencies by checking the applicable boxes below:**

CAG, Other

**Please indicate your source of funding;:**

Kelowna General Hospital, Interior Health

**Disclosure of Interest:**

None Declared

